# A ligase-based toolbox for research and diagnostics in molecular medicine

**DOI:** 10.1093/nar/gkag551

**Published:** 2026-06-02

**Authors:** Ulf Landegren

**Affiliations:** Molecular Tools, Department of Immunology, Genetics & Pathology, Science for Life Laboratory, Uppsala University, Se-75185, Uppsala, Sweden

## Abstract

New approaches for molecular analysis continuously open new vistas in molecular medicine. Our lab has built a series of molecular detection techniques based on enzymatic ligation of synthetic oligonucleotides as versatile tools to gain new molecular insights. These fundamental techniques continue to yield new means for specific, high-throughput analyses of nucleic acids and proteins in contexts of relevance for molecular medicine. The combined potential for vast multiplexing and low sample consumption renders the assays described herein attractive as a basis for AI-assisted model building in medicine. Accordingly, this overview is aimed to present a ligase-based molecular toolbox to choose from in addressing present and upcoming analytical needs.

## Graphical Abstract

**Graphical Abstract:**
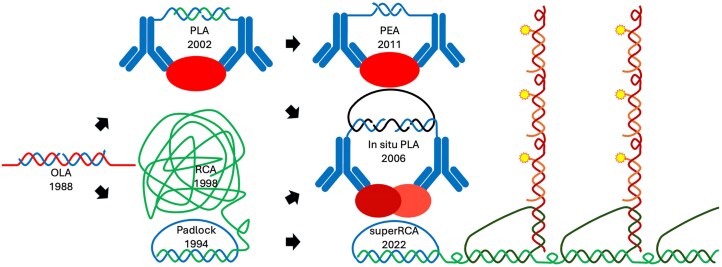

## Introduction

In the 1800s, Rudolf Virchow brought a change of perspective to medicine, from an earlier focus on the symptoms of disease and the affected organs, to instead considering what type of cells that might be affected by a disease and how, by way of cellular pathology [1]. With the advent of greatly improved molecular understanding of life processes, this cellular perspective has given way to a molecular view of medicine, pioneered by Linus Pauling with his 1949 identification of sickle cell anemia as a molecular disease [2]. The increasing availability of comprehensive information about the molecular composition of humans through the human genome project and subsequent efforts, along with a vast and increasing range of analytic technologies now contribute to a rapidly growing understanding of the molecular basis of both health and disease. A fundamental aim is to promote understanding, diagnostics, and treatment of disease by investigating what molecules may be associated with the disease processes.

Our lab at Uppsala University has an overarching ambition to contribute to this development by constructing tools and research strategies for molecular medicine. My purpose here is to focus on molecular tools developed by many talented students in my laboratory over almost 40 years, to illustrate previous and ongoing work to address needs in molecular medicine. For a timeline see the picture abstract. This paper makes no effort to summarize the vast literature on further developments and applications of these technologies in other labs. The paper does, however, mention some companies that I have economic interest in. I will indicate how we have adapted the basic tools to address a variety of analytical needs, hoping that the techniques will continue to enable new approaches to solve problems in molecular medicine and beyond.

A common theme for molecular reactions developed in our lab, as well as in many others, is to combine synthetic DNA strands with enzymes acting on DNA or RNA. While for example polymerase chain reaction (PCR) combines oligonucleotides with a DNA polymerase, and the Cas9 protein is central in gene editing, in work from our lab DNA ligases that join strands of DNA have a central role. In the following, I will describe our two principal approaches for ligase-mediated detection of nucleic acid sequences and of proteins, respectively, while illustrating some variants and applications of these techniques, with a perspective toward future directions.

### DNA ligation-based detection of nucleic acid sequences

With the increasing availability of human genome sequence information in the late 1980s it became apparent that a molecular basis for hereditary disease, which had for a long time been imperfectly gleaned in particular from twin studies, might be pinpointed along the chromosomes at least at modest resolution. This was shown to be possible by typing sufficient numbers of genetic variants in large-enough groups of patients and controls—what later became known as genome-wide association studies or GWAS [3, 4]. I developed a first ligase-based DNA detection technique to address this emerging need to monitor genetic polymorphisms.

#### The oligonucleotide ligation assay

**Figure 1.**
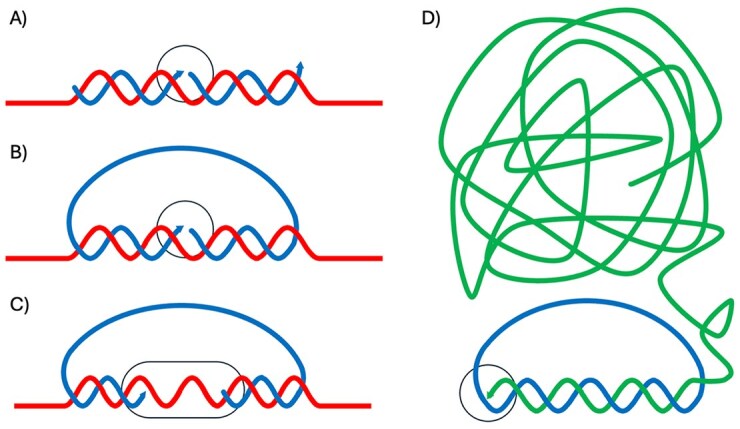
DNA ligation assays. (**A**) In the OLA, pairs of oligonucleotide probes (blue) hybridize next to each other to a target nucleic acid sequence in a sample (red). This allows the oligonucleotides to be joined by enzymatic ligation, provided that they are correctly matched. (**B**) Padlock probes are linear DNA probes that can be converted to DNA circles when their target-complementary end sequences are joined by ligation, templated by a target DNA or RNA sequence. (**C**) Gap-fill padlock probes can yield DNA circles by ligation only after missing segments between the 5′ and 3′ ends are filled in with preformed oligonucleotides or by a DNA polymerase copying part of the target sequence. (**D**) Once a padlock probe has been converted to a DNA circle, it can template a rolling-circle amplification (RCA) reaction by a DNA polymerase, continuously copying the endless template to generate a DNA strand composed of complements of the starting DNA circle (green). In the figure probe sequences are blue, targets are red, and the RCA product is green. The sites of enzymatic activity are indicated by black circles.

In the late 1980s during my postdoc with Lee Hood at Caltech, I established the oligonucleotide ligation assay (OLA) for genotyping purposes [5] (Fig. 1A). As described herein, several further techniques have been built on this simple mechanism to create new nucleic acid sequences by joining preexisting ones. In OLA, pairs of oligonucleotides are designed so that they can be joined by ligation, creating the missing phosphodiester bond, if they hybridize in juxtaposition to a target sequence present in a sample. The assay can discriminate between sequence variants that may differ in just a single nucleotide position at the site of ligation, as ligation of mismatched probes is inhibited because of stringent substrate requirements by the ligase.

#### Padlock probes

In view of the increasing need for high-throughput genotyping, we later modified the OLA technique by constructing padlock probes [6]. My then PhD student Mats Nilsson had a central role in this early and much subsequent work with padlock probes. These probes are linear oligonucleotides with 5′ and 3′ end segments designed to hybridize next to each other on a target sequence (Fig. 1B). Upon hybridization, the padlock probes may be converted to DNA circles by a ligase, optionally after a gap of one or more nucleotides is first filled in [7] (Fig. 1C). Just like PCR, padlock probes require joint target recognition by two sequences of 20 or so nucleotides, ensuring sufficient specificity to detect single targets among the sequence of the around 10^10^ or so deoxyribonucleotides that can be found in each human cell nucleus.

In multiplex target detection assays by either PCR or OLA, any pair of probes can give rise to reaction products. When greater numbers of sequences are targeted, the increasing number of possible probe pairs rapidly increases risks of cross-reactivity. In contrast, padlock probes only yield circular—detectable—reaction products when the sequences at the 5′ and 3′ ends of the same probe have engaged their target sequences, since joining two different probes fails to produce DNA circles and are therefore not detected in procedures that depend on the formation of circular DNA strands. This requirement for intramolecular dual-recognition is key for multiplexing specific DNA detection reactions. In fact, human genomic DNA samples are now being successfully probed using padlock probes for >200 000 single-copy sequence variants in parallel in a commercial product [8]. That number of parallel detection reactions is vastly greater than what is possible by either PCR or OLA.

Reacted padlock probes also have the interesting property that upon ligation they become wound around their target DNA strands because of the helicity of double-stranded DNA. Circularized padlock probes are thus stably locked on their targets, hence the name. Reacted probes therefore survive denaturing washes that remove unreacted probes. It is sometimes practical to also dispense with unreacted padlock probes by treatment with exonucleases that preserve only the reacted probes as they lack free ends.

Padlock probes are sometimes referred to as molecular inversion probes [7], because of the potential to subject the reacted probes to circular permutation, linearizing circularized probes by cleaving them at a site outside the target-complementary region. This creates linear amplicons for PCR that strictly depend on a prior intramolecular, dual-recognition reaction by padlock probes, enabling high multiplexing.

#### Rolling-circle amplification

The circular nature of reacted padlock probes, having no free ends, renders them suitable as templates for local signal amplification via RCA (Fig. 1D)—a mechanism well known from replication of some virus and plasmids [9]. As mentioned, DNA circle formation by padlock probes is a highly specific reaction, even in high multiplex. It is attractive to combine this reaction with the exquisitely DNA circle-dependent RCA mechanism for local accumulation of long DNA strands, as first shown by Lizardi *et al*. [10]. For each starting DNA circle, the Phi29 polymerase generates a strand containing around one thousand complements of a 100-nt circularized padlock probe in a 1-h reaction. The combination of padlock probing and RCA has been varied extensively [10–12].

Localized padlock probing with RCA results in discrete, prominent RCA products for each detected nucleic acid target, by virtue of the local accumulation of DNA strands with many complements of the DNA circle. The spot-like staining pattern with locally repeated barcode sequences can be read out by combinatorial labeling. For even higher throughput, Mats Nilsson and colleagues have demonstrated the possibility to sequentially interrogate large numbers of RCA products for padlock probe-based *in situ* analysis of transcripts in tissues sections [13].

The concept of padlock probes has been further generalized. It may for example be useful to create DNA circles from two or more DNA segments, guiding their ligation by specific oligonucleotides, for instance in the *in situ* proximity ligation assay (isPLA) described below. It is sometimes practical to reverse the roles of the DNA strand to be circularized and the ligation template. For this purpose, a synthetic oligonucleotide—a selector probe—can be designed to serve as template for the circularization of one of the strands of a particular restriction fragment directly from a DNA sample without any *in vitro* copying [14]. This can serve to avoid *in vitro* replication errors, and to preserve the methylation status of nucleotide residues in the DNA circles.

#### Circle-to-circle amplification

**Figure 2.**
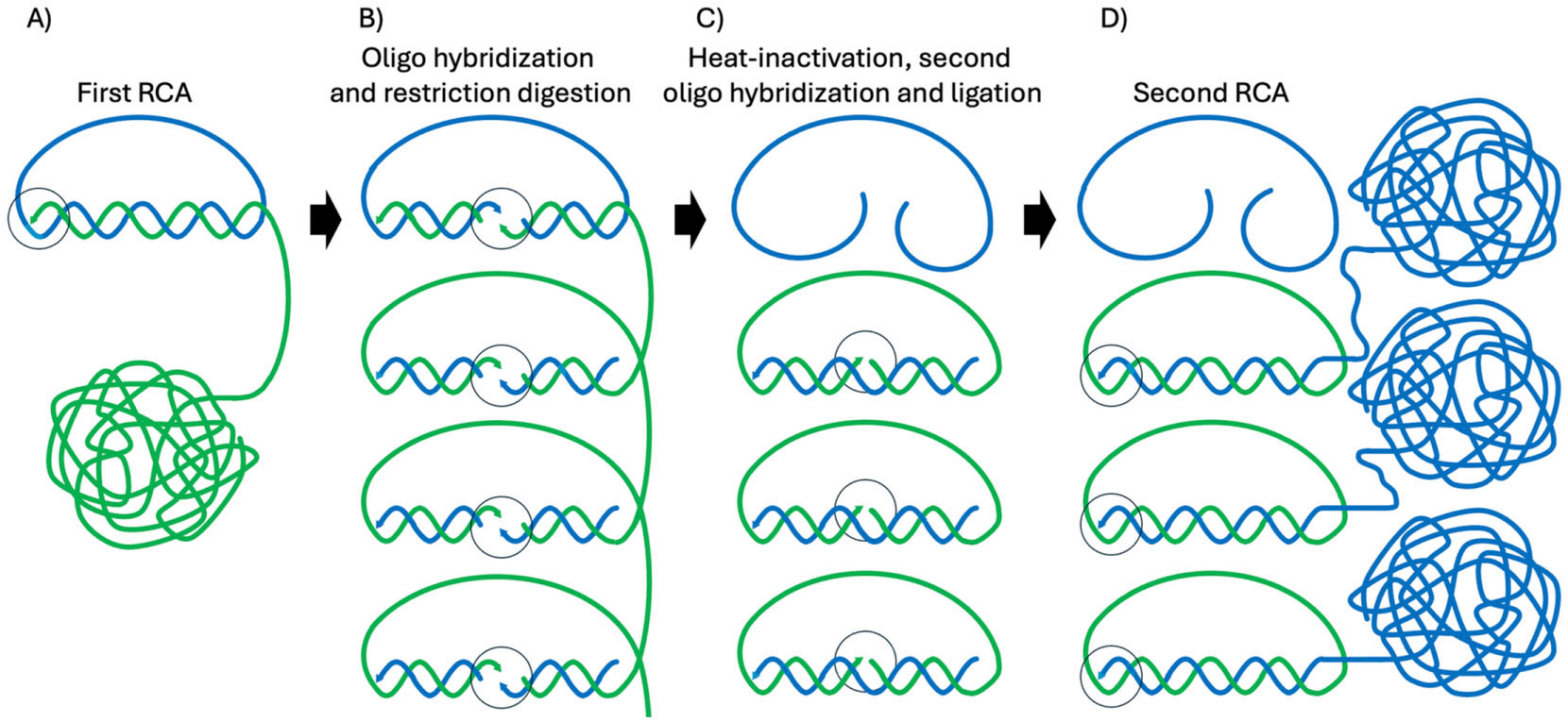
C2CA. (**A**) A DNA circle (blue) serves as template for replication, generating an RCA product (green), composed of multiple complements of the DNA circle. (**B**) Oligonucleotides (blue) are added that hybridize to the RCA products, directing digestion by an added restriction enzyme. (**C**) A heating step inactivates the restriction enzyme and dissociates the restricted oligonucleotides. When the temperature is lowered, intact oligonucleotides guide ligase-mediated joining of the ends of the monomerized RCA product. (**D**) The oligonucleotide reagents directing restriction and then ligation are next ready to prime replication, this time generating RCA products of a polarity opposite to that from the first-generation RCA. The reaction may be repeated. The two polarities of the sequences are indicated by the colors blue and green in the figure, and the sites of enzyme activity are indicated by black circles.

While PCR provides exponential amplification of copies of target molecules, RCA is limited to generating a single strand composed of copies of complements of each starting DNA circles. Accordingly, the reaction is suitable for localized and/or digital detection. It is possible, however, to achieve greater than linear i.e. polynomial amplification of the sequence information in DNA circles. In the circle-to-circle amplification (C2CA) technique, RCA products are monomerized by restriction digestion, guided by an oligonucleotide (Fig. 2) [15, 16]. Upon heat inactivation of the restriction enzyme and subsequent lowering of the temperature, digested oligonucleotides are replaced by intact ones, hybridizing to the monomerized RCA products and allowing these to form DNA circles catalyzed by a ligase. The same oligonucleotides are next ready to prime RCA templated by the circularized monomers, generating products of a polarity opposite to that of the first RCA products, for amplification at an approximately squared rate over time. If desired, the procedure may be repeated.

From a starting DNA circle, C2CA can thus generate amplified circular monomers, linear monomers, or multimers of either polarity, for divers analytical or preparative purposes.

Next, we also sought to develop a procedure where each starting DNA circle would result in precisely one large cluster of DNA sequences for sensitive digital detection and other purposes.

#### Super rolling-circle amplification

It is a recurring challenge in molecular medicine to study rare molecular event to identify disease at its roots by observing signs of disease as early as possible, before full-blown pathology may develop at some later time. This requires molecular tools that must be (i) very efficient in order not to miss rare target molecules, (ii) highly selective to avoid cross-reactivity for the vast excess of other, perhaps similar molecules that are likely to be present in a sample, and (iii) they should generate unmistakable reaction products to avoid the risk that true signals might be confused with any nonspecific background. Regular PCR or nested versions thereof as well as digital PCR can be used for detecting rare nucleic acid sequences, and sufficiently deep sequencing of barcoded DNA can provide very high sensitivity, but there has remained a need for alternative approaches.

**Figure 3.**
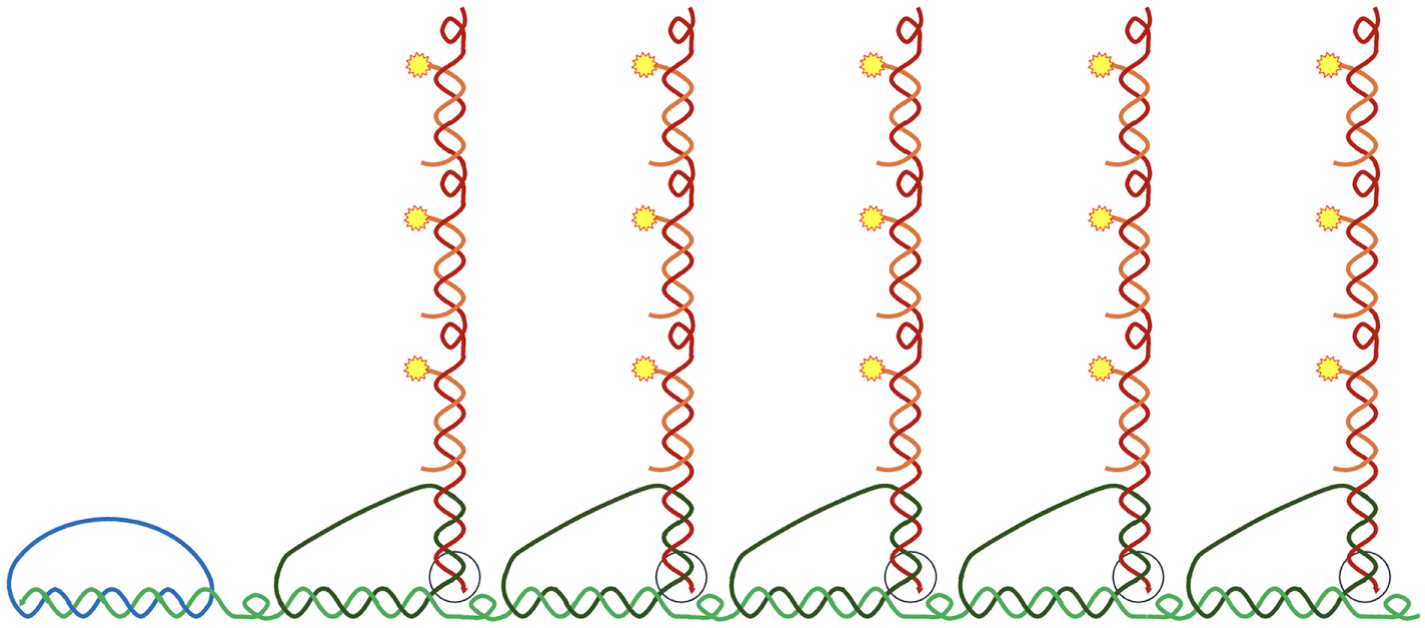
In the superRCA technique, a circularized DNA sequence (blue) is copied by RCA, generating an RCA product (green). The repeated sequences in the RCA product are then interrogated with padlock probes (black) to investigate which sequence variant that is present in many copies in this RCA product. Successfully circularized secondary padlock probes can in turn be replicated, generating secondary RCA products (dark brown). For each starting DNA circle the process results in one large DNA cluster, which may be labeled by hybridizing tag sequence-specific fluorescent oligonucleotide probes (light brown), and be distinguished and counted by flow cytometry or microscopy. The sites of extension by a DNA polymerase for the final RCA are indicated by black circles.

During his PhD in my lab Lei Chen developed a padlock probe-based method that can confer a very high selectivity for DNA sequence variants, through a technique we call superRCA (Fig. 3) [17]. Unlike the exponential multiplication of copies of target sequences by PCR, this technique produces for each detected target molecule one single, very prominent reaction product. This is accomplished through two or more consecutive generations of RCA that can also serve to enhance both specificity and visibility.

In this manner, a first RCA reaction from a starting DNA circle produces one strand composed of a thousand complements of the DNA circle. Once the monomers of the RCA product have been recognized by padlock probes, reacted probes can be subjected to another 1000-fold amplification, all contained in a single, large cluster of DNA sequences. This superRCA method can be applied to measure even very low proportions of tumor-specific DNA in blood, by first capturing in DNA circles sequences known to be somatically mutated if they are derived from a patient’s malignant cells. This target capture can be achieved using gap-fill padlock probes to generate DNA circles that incorporate DNA segments known to be mutated only in tumor tissue. Alternatively, sequences of interest can be amplified through a few PCR cycles before amplified DNA strands are converted to DNA circles by ligation, guided by an oligonucleotide that hybridizes to both ends of a PCR strand.

The circularized DNA molecules are replicated by RCA, generating strands that each contain hundreds or more copies of either the mutant or the wildtype sequence, depending on which variant of the target sequence that was incorporated in each starting DNA circle. Next, padlock probes specific for either mutant or wildtype sequence variants are added to genotype the RCA products. This step takes advantage of the substrate specificity of the ligation reaction to discriminate against mismatched probes. However, since the target molecules are concatemers, each containing many copies of either the wildtype or the mutant target sequence, any occasional mistyping by padlock probing can be ignored against a majority of correct reactions. This “majority voting” mechanism results in essentially error-free genotyping of the starting DNA circles, allowing for detection of exceedingly rare sequence variants.

Once reacted, the genotyping padlock probes that have become wound around the first-generation RCA products are in turn amplified in a secondary RCA. The procedure generates, for each starting DNA circle, one large bundle of DNA that can reach gigaDaltons in molecular weight, and with dimensions of a small cell. The many repeated complements of the reacted genotyping padlock probes can be detected using e.g. fluorophore-labeled oligonucleotides, allowing the products to be distinguished and individually counted in solution using generally available flow cytometers, or by microscopy. The superRCA procedure involves an easily automated series of additions of reagents and incubations over the span of a few hours, but no washes or separations.

We have demonstrated that point mutations present in genomic DNA from as little as one cell out of 100 000 can be readily detected using the superRCA method. This corresponds to the very tall order of correctly genotyping one single nucleotide for every 10^15^ nucleotides in a DNA sample. The superRCA technique can provide a key tool for precision medicine by aiding in selecting and adjusting therapy for cancer patients, or to monitor them for recurrence of disease. The assays can be read out using an installed base of flow cytometers at hospitals and research labs. For leukemias abundant DNA is available in nucleated cells in blood or bone marrow samples. For solid tumors, the assay sensitivity is restricted by the limited amounts of cell-free DNA released by the tumor to blood plasma. Sensitivity may be increased, however, by simultaneously targeting several tumor-specific mutations, as the assay lends itself for multiplex analyses. More generally, the superRCA technique provides a highly specific means to distinguish and enumerate any single or large set of molecules that may be represented by DNA circles. Many other formats for and applications of the assay can be anticipated. In preliminary work, we have shown that the superRCA assay may be adapted *in situ* to visualize rare cells harboring mutant transcripts in tissue sections. superRCA assays can be an element of a general, important transition from estimating levels of molecules in the analogue domain to digitally evaluating and enumerating the molecules. Such assays may be conducted in solution as well as by mapping the spatial distribution of molecules in tissues. Our spin-out company Rarity Bioscience is producing superRCA assays for monitoring the presence of tumor-specific DNA in blood from patients with leukemia or solid cancer.

#### Some further industrial applications of DNA ligation for nucleic acid analyses

My students and I have founded a series of companies whose technologies build on aspects of the padlock probe concept in a loose sense, addressing a wide variety of analytic needs. ParAllele was an early multiplex genotyping company, cofounded with colleagues at Stanford and now part of Thermo Fisher. HaloGenomics developed a related intramolecular ligation technology for targeted genome sequencing in multiplex, and that company is now part of Agilent. As already mentioned, Rarity uses padlock probes for detecting rare mutant DNA sequences in liquid biopsies, while the company Readily develops point-of-care virus diagnostics by seamlessly combining padlock probing and RCA. Former students from the lab have also gone on to commercialize several variants of the padlock probe concept for applications such as noninvasive prenatal testing by Vanadis, now part of Perkin Elmer. Padlock probe-based analysis with RCA for spatial transcriptomics was developed by Cartana, founded by Mats Nilsson and now acquired by 10X Genomics, and permit localized detection and counting of thousands of specific transcripts *in situ*.

Next, we will see how DNA ligation reactions can also prove of value for antibody-based protein analyses.

### DNA-assisted protein detection

While DNA analyses provide insights in the genetic potential of cells and organisms, protein measurements offer insights in the current state-of-affairs. Affinity-based protein assays were for a long time limited to either sandwich antibody assays first published in 1967 [18], using pairwise combinations of capture and detection antibodies for demanding analyses of one protein species at a time, or assays where binding by single antibodies per protein were used at lower accuracy in single or in multiplex formats. As described hereunder DNA-assisted protein assays have created entirely new opportunities.

#### Solution-phase proximity assays

**Figure 4.**
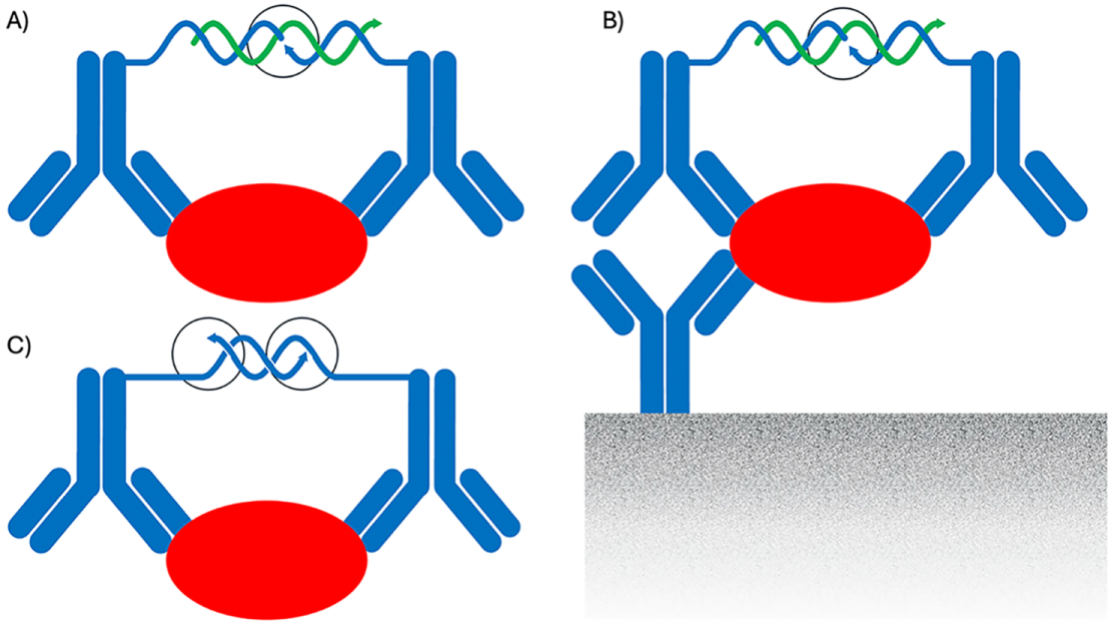
Proximity assays. (**A**) In PLA, oligonucleotides (blue) attached to a pair of antibodies (blue) that have bound the same target protein can be joined by ligation, guided by an added oligonucleotide, after the reaction has been diluted to minimize chance proximity between the antibody–oligonucleotide conjugates. The ligation products can be recorded via PCR or DNA sequencing as surrogate markers for the detected proteins. (**B**) In solid-phase PLA the target proteins are first captured on a solid support (gray gradient) before addition of the PLA reagents. This form of the assay admits larger sample volumes and removal of excess reagents by washes before ligation and recording the outcome of the reactions. (**C**) In the related proximity extension assays samples are incubated with pairs of antibodies conjugated to oligonucleotides with short complementary segments at their 3′ ends. Upon diluting the reactions, a DNA polymerase can extend oligonucleotides, attached to nearby antibody pairs, creating amplifiable reporter DNA products. The sites of enzyme activity are indicated by black circles.

In the mid-1990s, our lab set out to combine powerful molecular genetic techniques with affinity-based protein detection in proximity ligation assay (PLA). We connected oligonucleotides to pairs of protein-binding reagents, such that the oligonucleotides could be joined by ligation upon proximal binding of pairs of binder-DNA conjugates to the same protein targets. This PLA, first published in 2002 (Fig. 4A) [19, 20], creates DNA amplicons with sequence element from two oligonucleotides attached to pairs of DNA aptamers or antibodies, as evidence of successful protein detection. Proximity ligation reactions can be read out either using real-time PCR or by high-throughput sequencing the amplicons that arise in the detection reactions. Among many talented PhD students, Simon Fredriksson has over many years had a crucial role in this work.

PLA share the enhanced specificity of dual-recognition sandwich immune assays, in that only proteins recognized by a pair of antibody–oligonucleotide conjugates can be detected, thus ignoring cross-reactive binding to irrelevant molecules provided this cross-reactivity is not shared by the antibody pair. Proximity assays differ from regular sandwich assays, however, in that by design only DNA ligation or extension reactions between the intended pairs of antibody–oligonucleotide conjugates give rise to detectable reporter molecules even among large sets of reagents, thus allowing analyses in high multiplex without risking increasing numbers of nonspecific reactions between unintended reagent pairs. The assays are typically performed entirely in solution-phase. Nonspecific background from reagents that are in proximity by chance is reduced by a simple dilution step, instead of through washes of molecules captured on supports as commonly done in conventional sandwich assays. The possibility to amplify the DNA strands that record detection reactions ensures high sensitivity, provided nonspecific background is kept to a minimum, thereby allowing the use of sample volumes of a single microliter or less for multiplex reactions. Nonetheless, solid-phase versions of the PLA reactions, where target molecules are captured by a third antibody immobilized on a support, may offer advantages for demanding applications because (i) larger sample volumes can be screened for dilute target proteins, (ii) excess reagents, potentially creating nonspecific signals, can be removed by washes, (iii) the requirement for recognition by not just pairs but trios of antibodies further reduces risks of accidental cross-reactivity for irrelevant target molecules, and (iv) any background from nonspecifically bound reagents is reduced, since unlike in sandwich immune assays, no single reagent alone can produce detectable signals, only pairs of target-binding antibodies (Fig. 4B) [21]. For a recent review of PLA see [22].

#### Proximity extension assay

In the proximity assays developed and marketed by our spin-out Olink Proteomics, now part of Thermo Fisher, a solution-phase DNA polymerase-based extension reaction is used in lieu of DNA ligation to similar effect in multiplex proximity extension assays (PEA; Fig. 4C). Presently, >5000 proteins are analyzed in 2 µl sample aliquots, with read-out via next-generation sequencing. Billions of protein datapoints are currently being generated using this PEA in a wide range of biomedical contexts. Olink’s PEA analyses, along with Somalogic’s conceptually different Somascan technique [23], have vastly improved opportunities to monitor the protein composition of patient samples. It is gratifying to watch the rapidly growing literature revealing protein patterns with striking correlation to diseases, discovered using these multiplex assays. The assays have become an important means to better understand the roles of proteins during drug development, and the patterns accurately reflecting both chronic and acute disease states promise important applications in clinical diagnostics.

The proximity reaction mechanism has been varied extensively by us and also by many others to address different requirements. For example, the companies Alamar and Spear have both modified proximity assays for additional sensitivity by capture and washes [24] or by a requirement for two consecutive enzymatic reaction steps [25], respectively. Both modifications serve to minimize nonspecific background, arising from transient target-independent proximity reactions.

#### Measurement of antibody reactivity

We note that plasma samples also contain antibodies with specificity for wide ranges of antigens that the sample donors are immune toward, and that can be of diagnostic interest. Measurements of the repertoires of antibody specificities in plasma samples offer salient insights in what infections an individual has experienced, and his or her state of immunity against vaccines. Individuals may also carry antibodies against self-antigens as diagnostic manifestations of autoimmunity or risks thereof, sometimes predating clinical onset of disease by decades. A further growing interest concerns antibody repertoires in cancer patients toward neoantigens, i.e. somatically mutated tumor proteins, some of which may contribute to the malignant phenotype or immune responses. Neoantigens may serve as immunization targets in cancer, and they could also assume a diagnostic role.

**Figure 5.**
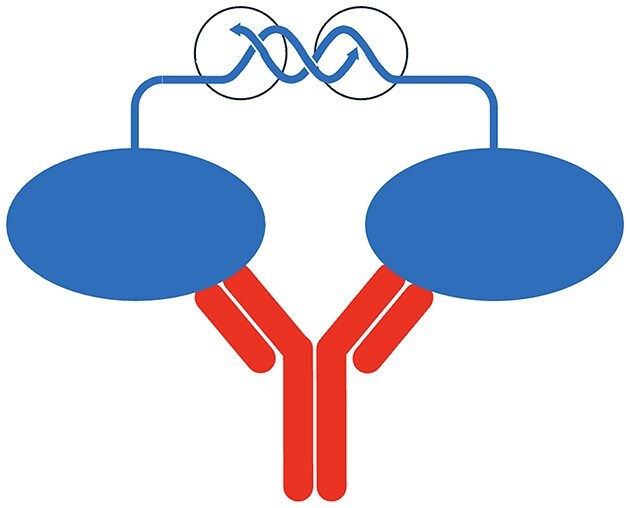
In AbPEA, the amounts of antibodies directed against specific antigens can be measured in assays that may be described as inverted PEA reactions. Instead of conjugating oligonucleotides to pairs of antigen-specific antibodies, the oligonucleotide pair (blue) is conjugated to two aliquots of an antigen preparation (blue ovals). The assay results in proximity-based extension products in proportion to the presence of antigen-specific antibodies (red) in the investigated sample, able to bring pairs of antigen molecules in proximity. The sites of enzyme activity are indicated by black circles.

To meet the need for high-throughput and high-performance measurement of antigen reactivity, my coworker Hongxing Zhao reversed the PEA mechanism by attaching oligonucleotides to pairs of identical antigen molecules (Fig. 5) [26]. A related approach was reported by Tsai *et al*. [27]. Antigen-specific antibodies in plasma samples can bring such pairs of antigen–oligonucleotide conjugates in proximity by virtue of their dual or greater valency. Following the PEA approach, reactions where samples have been incubated with antigen–oligonucleotide conjugates are diluted to reduce nonspecific background before a DNA polymerase is added to generate amplicons that represent the specific antibodies. As little as one millionth of a microliter of plasma can suffice for detecting reactivity toward an autoimmune target in systemic lupus erythematosus, in this antibody-PEA or AbPEA reaction. Just like in regular PEA, the assays can be performed without washes or separations and are then read out by real-time PCR or in higher multiplex by sequencing. We have also demonstrated that reactivity to specific antigens was readily detected in both wet and dry samples of either blood or saliva [28], simplifying sample collection. As described below, we saw a need for further improved blood sample collection.

#### Enhanced blood sample collection

The new opportunities for informative multiplex measurements of proteins, antibodies, and other biomarkers in either wet or dry plasma or other bodily fluids using ligase- or polymerase-mediated techniques are generating valuable information on states of health, accurately reflecting a wide variety of pathologies and their dynamic course over time. A recurring pattern in multiplex plasma protein analyses is that each person maintains his or her own profile of biomarkers that remain relatively stable over time, but that factors such as advancing age and emerging diseases characteristically alter these profiles [29]. The cost of molecular analyses will continue to decrease. It is therefore a high priority to now begin collecting consecutive blood samples, both to help evaluate whether an individual deviates from his or her own normal range, and to create a knowledge base of marker constellations that predict onset or changes to the course of disease.

Because of the limited sample volumes required for modern analyses, we explored dried blood spots from finger pricks as a source of blood samples. My then PhD student Johan Björkesten ascertained that a 1-mm^2^ cut from a blood drop dried on paper suffices for recording protein levels using an Olink panel for 92 proteins [30]. This provides a means to construct at low cost extensive biobanks with many consecutive samples from large numbers of donors. Since whole blood is not normally used for analyses, we went on to develop a simple donor-centric sample collection device where blood cells and plasma from finger pricks are instantly separated by filtration before drying. Drops of blood are placed on two membranes that become pressed against each other upon opening a booklet. Blood cells stay on the top membrane, while plasma is transported in a matter of seconds by capillary action to the lower membrane brought in close contact. The dried membranes can be sent by regular mail, stored in a compact fashion with limited refrigeration. Multiple portions may be cut from each sample by a lab robot to be processed for analysis of proteins, antibodies, nucleic acids, etc. (Ekholm *et al*. in preparation). The rapid separation of cells and plasma also ensures accurate measurement of plasma levels of components such as proteins, without influence by variable hematocrit. The separation is also helpful for measuring components that may be present in both blood cells and plasma. For example, plasma levels of potassium ions are diagnostically interesting in cardiovascular disease, and can be measured despite the much higher levels in blood cells [31]. The devices for separate collection of dry blood cells and plasma are made available by our spin-out SampleFacts.

#### Localized proximity assays

As discussed above, the ready accessibility of peripheral blood permeating the body renders it attractive as a source of molecular information sampled from the whole body. Nonetheless, it is important also to observe molecular processes where they occur in tissues among and within the myriad interacting cells. Accordingly, spatial biology is assuming increasing interest. While techniques for solution-phase protein detection have undergone dramatic development as exemplified above, to this day most affinity-based *in situ* protein detection assays still rely on simple staining with one or more fluorophore- or enzyme-labeled antibodies as first published by Coons *et al*. already in 1941 [32].

**Figure 6.**
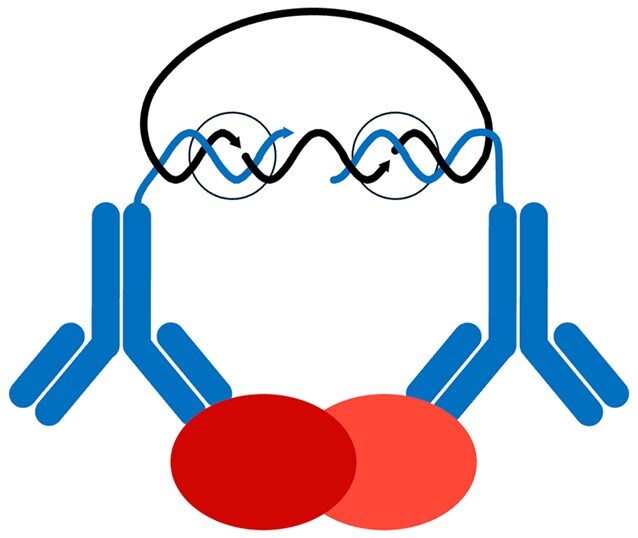
The *in situ* PLA technique allows locally amplified detection of proteins *in situ* upon pairwise binding by oligonucleotide-conjugated antibodies in proximity. Two antibody–oligonucleotide conjugates (blue) that have bound their targets in proximity (here a pair of dark and light red ovals) guide the formation of a circular DNA strand (black), which is then locally amplified by RCA for enhanced visibility. The requirement for binding by two antibodies helps to avoid background signals due to cross-reactivity by one or the other of the two antibodies, and it also provides an opportunity to reveal interacting or posttranslationally modified proteins, reflecting cellular activity states. The sites of enzyme activity are indicated by black circles.

To enhance the specificity of localized protein detection, my postdocs Ola Söderberg and Mats Gullberg adapted the proximity assay concept, using pairs of oligonucleotide-conjugated antibodies to generate circular DNA strands for localized protein detection via RCA by isPLA (Fig. 6) [33]. Just as for detection of proteins in solution by for instance ELISA or PEA, the requirement for target recognition by pairs of antibodies *in situ* enhances specificity by ignoring any cross-reactive binding not shared by the antibody pair. isPLA also provides a valuable opportunity to reveal the location not just of individual proteins but also where pairs of proteins have formed or dissolved interactions, reflecting acts of cellular signaling. The antibody pairs can thus confer important information about protein activity states by targeting pairs of proteins in dynamic interactions. Similarly, posttranslational modifications of specific proteins can be analyzed by devoting one antibody to identify the protein while another antibody serves to detect the modification. The mapping of protein activity states in cells and tissues by isPLA can be read out via microscopy or flow cytometry. The isPLA analyses can offer previously unavailable views of protein activity states in disease in cells and tissues and in response to therapy, supporting e.g. drug development or selection of optimal treatment for individual patients.

Similar to solution-phase PLA reactions, the isPLA reagents are constrained so that only the appropriate pairs of oligonucleotide-conjugated antibodies in proximal binding to targets can give rise to amplifiable DNA circles. Accordingly, isPLA, just like padlock probes and solution-phase PLA/PEA, can be performed in multiplex without rapidly increasing nonspecific reactions from noncognate pairs of antibody–oligonucleotide conjugates. As discussed for localized padlock probing of nucleic acid target sequences with RCA, the spot-like staining pattern lends itself for multiplex readout by reading a barcode sequence present in each RCA product. The isPLA technique is being commercialized by our spin-out Navinci Diagnostics and under license to Sigma Aldrich as Duolink assays.

Approaching the end of this report it is relevant to ask what may the future hold for molecular technologies? We can expect many things quite unpredictable even in the short term no doubt, but other things will develop as extrapolations of the present.

### Concluding remarks

As discussed herein, a few basic molecular tools can go a long way. The combination of synthetic oligonucleotides with DNA-specific enzymes, here mainly DNA ligases, can be used to construct molecular tools to address analytical needs as these are being identified. Large sets of plasma proteins and rare mutant DNA in blood from cancer patients or in tissue samples are examples of biomarkers that convey crucial medical information. This is also true for activity states of proteins in intra- and intercellular communication that can inform tumor therapy targeting upregulated sgnaling cascades. Infectious medicine is yet another large area with many unmet needs where ligase-based tools can play important roles. It remains a pervasive challenge to identify previously unexplored classes of molecular biomarkers that can provide decisive informative, and secondarily to this, creating the tools to analyze these markers.

For some things, like genome sequences, reading out the total information content will increasingly make sense with the dwindling cost of sequencing, but other assays will remain more targeted and focusing only on small numbers of parameters that convert to easily evaluated algorithms, perhaps with yes/no output in clinical settings. Many new assays will find their way to the point of care at doctors’ offices or in people’s homes to allow individuals to manage their own health, including by repeatedly monitoring health parameters. This is in line with a strong tendency to bring healthcare out of hospitals and into people’s homes. On the other hand, high-throughput, high-performance assays at levels of nucleic acids and proteins from small sample aliquots are promising for the massive data generation that will be needed to build vast foundation models for AI-supported health care over coming years [34].

In short, tools for molecular medicine have come a long way, but the area is still in flux with continuous development of increasingly informative, efficient, and accessible means to measure the molecular basis of health and disease.
